# Comparison of chemical profiles between the root and aerial parts from three *Bupleurum* species based on a UHPLC-QTOF-MS metabolomics approach

**DOI:** 10.1186/s12906-017-1816-y

**Published:** 2017-06-12

**Authors:** Lin Zhu, Zhi-Tao Liang, Tao Yi, Yue Ma, Zhong-Zhen Zhao, Bao-Lin Guo, Jian-Ye Zhang, Hu-Biao Chen

**Affiliations:** 1 School of Chinese Medicine, Hong Kong Baptist University, Hong Kong, Special Administrative Region, People’s Republic of China; 2 Institute of Medicinal Plant Development, Chinese Academy of Medical Sciences, Peking Union Medical College, Beijing, 100094 China; 3 School of Pharmaceutical Sciences, Guangzhou Medical University, Guangzhou, 510182 China

**Keywords:** Bupleuri Radix, Chemical profiles, Medicinal parts, Metabolomics approach

## Abstract

**Background:**

Bupleuri Radix (Chaihu) represents one of the most successful and widely used herbal medicines in Asia for the treatment of many diseases such as inflammatory disorders and infectious diseases over the past 2000 years. In the Chinese Pharmacopoeia, Chaihu is recorded as the dried roots of *Bupleurum chinense* DC. and *B. scorzonerifolium* Willd. (Umbelliferae). However, the widespread demand for the herb has tended to far outstrip the supply. Whether the aerial parts, which account for 70 ~ 85% of the dry weights of *Bupleurum* species, could be used as an alternative for the root has become an important scientific issue for the sustainable utilization of *Bupleurum* species. On the other hand, in some areas including the southeast of China as well as in Spain, the aerial parts of *Bupleurum* species have already been used in the folk medications. Therefore, to clarify whether the root and aerial parts of *Bupleurum* species are “equivalent” in the types and quantities of chemical constituents which subsequently influence their biological activities and therapeutic effects is of great importance for both the rational and sustainable use of this herb.

**Methods:**

In the present study, the chemical profiles between the root and aerial parts of *Bupleurum* species from different species and collected from various locations were analyzed and compared by the ultra-high performance liquid chromatography quadrupole/time of flight-mass spectrometry (UHPLC-QTOF-MS).

**Results:**

A total of 56 peaks were identified in the root and/or aerial parts from different batches of *Bupleurum* species, by comparison of references standards or with those reported in the literature. Principal Component Analysis (PCA) was conducted for displaying the differentiating clustering between these two parts.

**Conclusion:**

The results disclosed the distinct variations between them, which indicated that the aerial parts could not be used as an alternative of root from a chemodiversity perspective. The differentiating markers resulted from the PCA analysis could also be utilized for the differentiation between them. Further validation of their biological differences is anticipated in the future study.

## Background

Bupleuri Radix (Chinese name: Chaihu) represents one of the most popular Traditional Chinese Medicines (TCM) over the past 2000 years. Its TCM indications include the treatment of influenza or common cold with fever, chills and fever from malaria, distending pain in the chest and menstrual disorders [1]. In the Chinese Pharmacopoeia, Chaihu is recorded as the dried roots of *Bupleurum chinense* DC. and *B. scorzonerifolium* Willd. (Umbelliferae) [1]. It is often found in clinical prescriptions and proprietary Chinese medicines, such as Xiao-Chaihu-Tang, Xiao-Yao-Wan, Jia-Wei-Xiao-Yao-Wan and Chai-Ling-Tang. In addition to the authentic species of Chaihu, there are more than 20 other species in the genus *Bupleurum* also habitually utilized as Chaihu in some local areas. Knowing the high demand for Bupleuri Radix and knowing the diversity of species that can be—both rightly and wrongly—used for this herb, the resources of Chaihu are very scare. Today, Chaihu from the species of *B. yinchowense* Shan et Y. Li has become mainstream in the market. [2] The species of *B. yinchowense* is abundantly distributed in the Northwest of China and is widely used in folk medicine for relieving fever, soothing liver and improving the symptoms of emotional instability such as depression, anxiety and phobia [3–5]. Additionally, in the Japanese Pharmacopoeia (16th edition), the official botanical origin of Bupleuri Radix (pronounced “saiko” in Japanese) is the roots of *B. falcatum* L [6]. Actually, *B. falcatum* is also commonly used in China and Korea [7]. In Japan, *B. falcatum* L is known for its therapeutic effects in the treatment of chronic hepatitis, auto-immune diseases and diabetes [8–11]. It is also used as an ingredient in herbal tea and traditional fermented beverages [8].

**Fig. 1:**
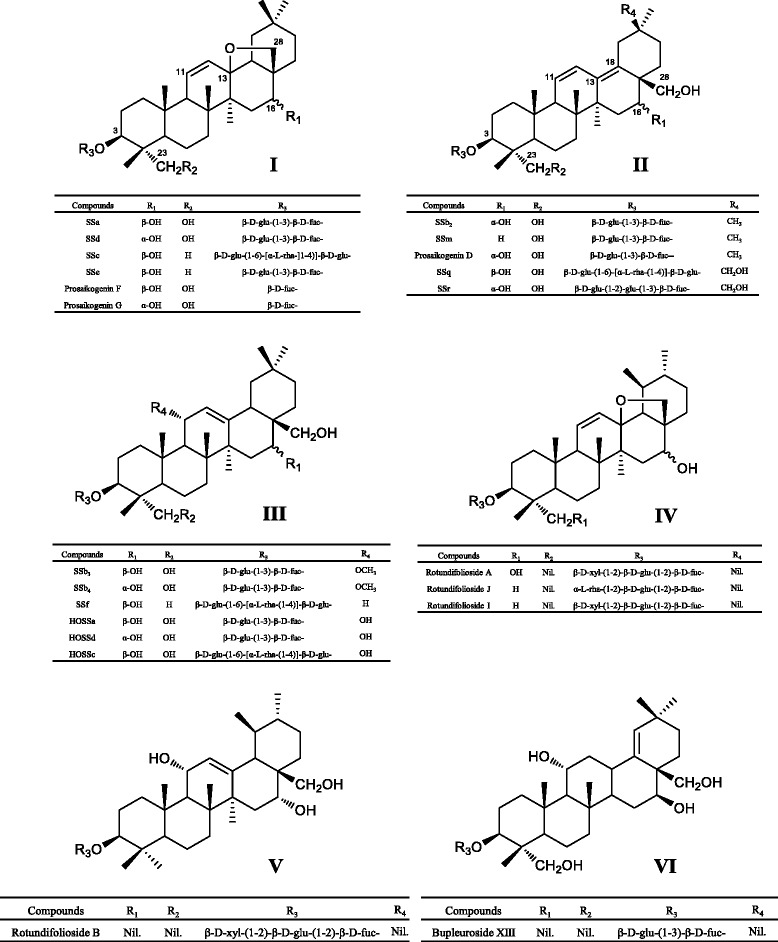
Chemical structures of six types (I-VI) of saikosaponins (SSs) in *Bupleurum*

Previous phytochemical studies on approximately 50 *Bupleurum* species led to the isolation and identification of almost 250 natural compounds from all major phytochemical classes, including mono- and sesquiterpenes (essential oils), triterpenoid glycosides (saikosaponins), sterols, lignans, flavonoid glycosides, coumarins, and polyacetylenes [12–14]. In addition, minor components, including phenylpropanoids, polysaccharides and a few alkaloids, have also been reported [12]. Among them, the saikosaponins (SSs) are acknowledged to be the principal bioactive components, which can be divided into six types on the basis of the aglycones: type I-VI (Fig. 1) [15, 16]. Flavonoids are another class of bioactive secondary metabolites present in all species of the genus *Bupleurum* [17]. Most flavonoids in the genus are derivatives of the flavonol aglycones kaempferol, isorhamnetin or quercetin [12].

Since Chaihu is very rare in nature, the amount of wild samples is not sufficient for commercial exploitation [18]. Although Chaihu have been widely cultivated nowadays, the widespread demand for the herb has still tended to far outstrip the supply [19, 20]. As the aerial part of *Bupleurum* species accounts for more than half of the whole plant, some areas in the southeast of China use the whole herb for the medication [21]. In Guangdong province of China, the root and aerial parts of *Bupleurum* species are sold separately for the folk use [22]. Besides, the aerial parts of *Bupleurum* species are used as a popular topical antiseptic and anti-inflammatory remedy in Spain [23]. Therefore, the short supply and perspective for sustainable utilization of Chaihu has stimulated the interest on comparing the “equivalence” of the root and aerial parts. Whether the root and aerial parts vary in the types and quantities of chemical constituents which subsequently influence their therapeutic effects, would determine whether the aerial parts of *Bupleurum* species could be used as a suitable substitute for the root. Additionally, whether Chaihu should be prescribed as root, aerial parts or whole plant of *Bupleurum* species was also needed to be clarified by scientific evidences, so as to prevent or avoid the misuse of this herb. To differentiate the crude materials from which medicinal parts used in proprietary Chinese medicines containing Chaihu is also significant for their quality control. Therefore, a comprehensive analysis of the chemical profiles is highly needed to be conducted for the two different parts.

The objective of the present study is to analyze and compare the chemical profiles between the root and aerial parts of *Bupleurum* species utilizing the ultra-high performance liquid chromatography quadrupole/time of flight-mass spectrometry (UHPLC-QTOF-MS). The authentic species of *B. chinense* as well as *B. yinchowense* and *B. falcatum* collected from different locations were investigated. The overall results provided comprehensive chemical comparison for the two different parts of *Bupleurum* species, which was anticipated to advise the possibilities of alternate use of these two parts.

## Methods

### Plant materials

**Fig. 2:**
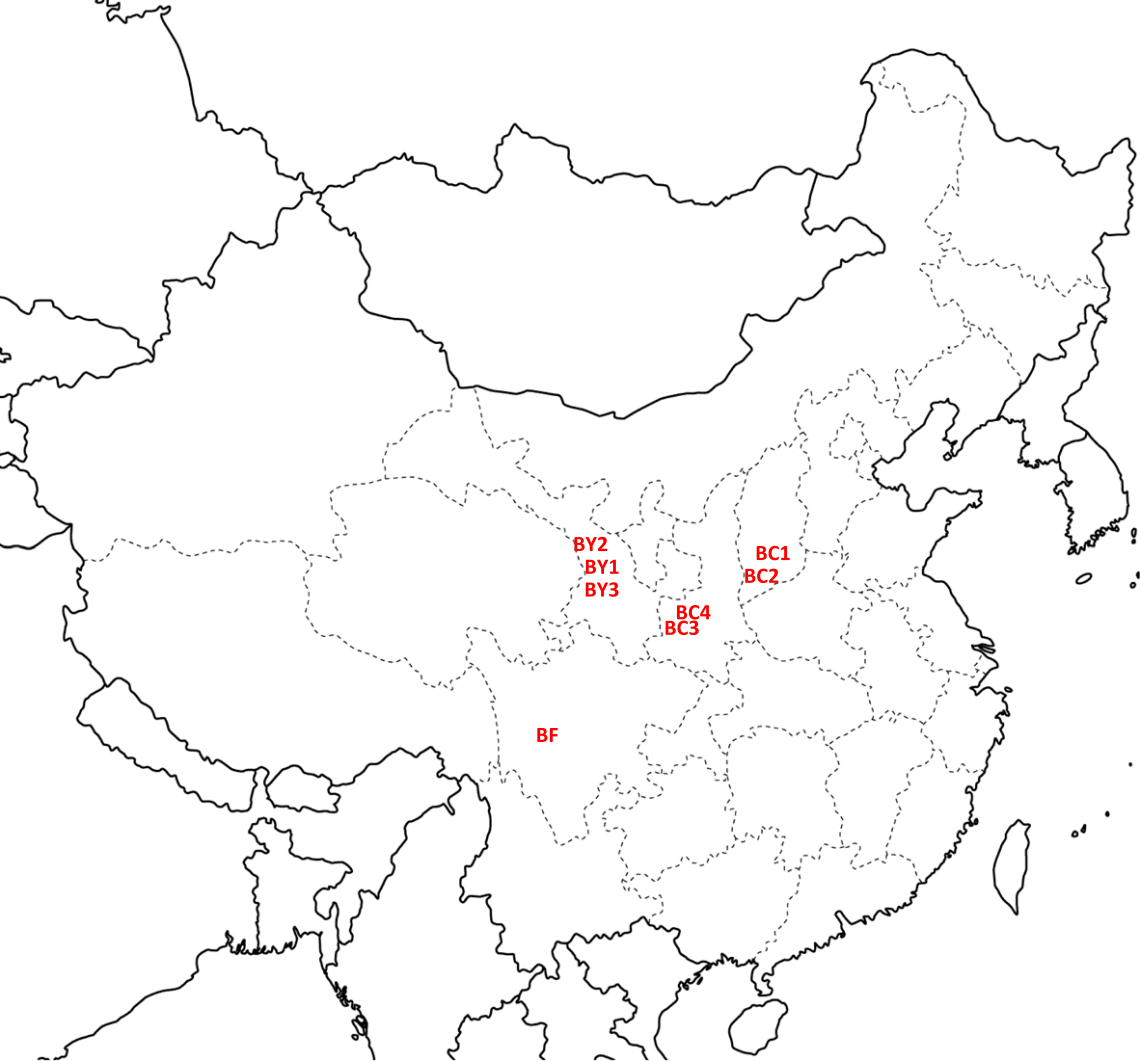
The locations of harvest of *Bupleurum* plants. (The blank map was obtained from a free Baidu library https://wenku.baidu.com/, and then the locations were marked on the map by the authors)

Eight batches of the whole plant including the root and aerial parts of *Bupleurum chinense* DC., *B. yinchowense* and *B. falcatum* were collected. Details of the sample are shown in Table 1 and Fig. 2. All the herbal samples were authenticated by Prof. Guo Baolin from the Institute of Medicinal Plant Development, Chinese Academy of Medical Sciences, Peking Union Medical College and deposited in the Bank of China (Hong Kong) Chinese Medicines Centre of Hong Kong Baptist University.

**Table 1:** Sample information for the analysis

No.	Species	Source (geographical location)	Collection time	Deposition numbers
BC1	*Bupleurum chinense* DC.	Wei Village, Fen Cheng town, Xiang fen County, Shanxi Province, cultivated for three years	2013–8-7	SX-0807
BC2	*Bupleurum chinense* DC.	Long Xing Village, Kao Lao town, Wan Rong County, Shanxi Province, cultivated for three years	2013–8-8	SX-0808
BC3	*Bupleurum chinense* DC.	Xiang Quan County, Chen Cang District, Bao Ji City, Shaanxi Province, wild	2013–9-4	SX-0917
BC4	*Bupleurum chinense* DC.	Xin Min County, Chen Cang District, Bao Ji City, Shaanxi Province, cultivated for three years	2013–9-4	SX-0918
BY1	*Bupleurum yinchowense* Shan et Y. Li	An Yi Town, Long Xi County, Gansu Province, cultivated for two years	2013–9-2	GS-0903
BY2	*Bupleurum yinchowense* Shan et Y. Li	Lu Ming Village, Wu Zhu Town, Wei Yuan County, Gansu Province, cultivated for two years	2013–9-2	GS-0905
BY3	*Bupleurum yinchowense* Shan et Y. Li	Da Hong Yan Village, San Fen Town, Zhang County, Gansu Province, cultivated for two years	2013–9-3	GS-0908
BF	*Bupleurum falcatum* L.	Han Yuan County, Sichuan Province, cultivated for one years	2013–9-5	SC-0919

### Chemicals and reagents

Chemical markers of saikosaponins a, c and d were purchased from Chengdu Must Bio-Technology Co., Ltd. (Chengdu, People’s Republic of China). The purities of all saikosaponins were determined to be higher than 98% by HPLC-DAD analysis. The solvents, acetonitrile and methanol, were of HPLC grade from E. Merck (Darmstadt, Germany). Formic acid with a purity of 96% was also of HPLC grade (Tedia, U.S.A.). Water was obtained from a Milli-Q water purification system (Millipore, Bedford, MA, U.S.A.).

### Preparation of sample solution

The dried roots and aerial parts of eight batches of samples involving *Bupleurum chinense*, *B. yinchowense* and *B. falcatum* from different growing areas were separated and grinded into homogeneous powders using liquid nitrogen. The dried powder (~0.1 g) was weighed accurately into a 15 mL microcentrifuge tube and was then extracted twice with 10 ml of methanol using an ultrasonicator (1875HTAG, CREST, U.S.A.) for 30 min at room temperature each time. After centrifugation at 3000 rpm for 10 min, the supernatant was transferred into a 25 ml volumetric flask and was adjusted to the volume with methanol. Finally, 1.0 mL extraction was transferred to 1.5 microcentrifuge tube and centrifuged again for 10 min at 12,000 rpm. An aliquot of 90 μl of supernatant was transferred to the glass inserts of 1.5 ml brown HPLC vials (Grace, HK) with plastic bottom springs (400 μl, Grace, UK) and stored at 4 °C pending for analysis.

Stock solutions of saikosaponins a, c and d were prepared individually in methanol. Working solutions were prepared by diluting the stock solutions with methanol to give final concentrations of 36, 10 and 36 μg/ml for saikosaponins a, c and d, respectively.

### UHPLC-QTOF-MS analysis

UHPLC–QTOF-MS analysis was performed on an Agilent 6540 ultra-high definition accurate mass quadrupole time-of-flight spec-trometer with UHPLC (UHPLC–QTOF-MS, Agilent Technologies, U.S.A.). A UPLC C_18_ analytical column (2.1 mm × 100 mm, I.D. 1.7 μm, ACQUITY UPLC®BEH, Waters, U.S.A.) was used for separation, coupled with a C_18_ pre-column (2.1 mm × 5 mm, I.D. 1.7 μm, Van-GuardTM BEH, Waters, U.S.A.) at room temperature of 20 °C. The mobile phase was a mixture of water (A) and acetonitrile (B), both containing 0.1% formic acid, with an optimized linear gradient elution as follows: 0–5 min, 10–35% B; 5–25 min, 35–55% B; 25–28 min, 55–85% B; 28–30 min, 85–100% B. The injection volume was 4 μL. The flow rate was set at 0.35 mL/min. The mass spectra were acquired in negative mode by scanning from 100 to 1700 in mass to charge ratio (*m/z*). The MS analysis was performed under the following operation parameters: dry gas temperature 300 °C, dry gas (N_2_) flow rate 5 L/min, nebulizer pressure 30 psi, Vcap 3000, nozzle voltage 500 V, and fragmentor voltage 200 V.

### Data analysis

Data analysis was performed on Agilent MassHunter Workstation software-Qualitative Analysis (version B.04.00, Build 4.0.479.5, Service Pack 3, Agilent Technologies, Inc. 2011). The acquired data in MassHunter Qualitative Analysis software were extracted using the molecular feature extraction (MFE) algorithm and imported into Mass Profier Professional (MPP) V.12.5 for principal component analysis (PCA) to display the difference between the root and aerial parts of eight herbal samples.

## Results and discussion

### Chemical profiling

**Fig. 3:**
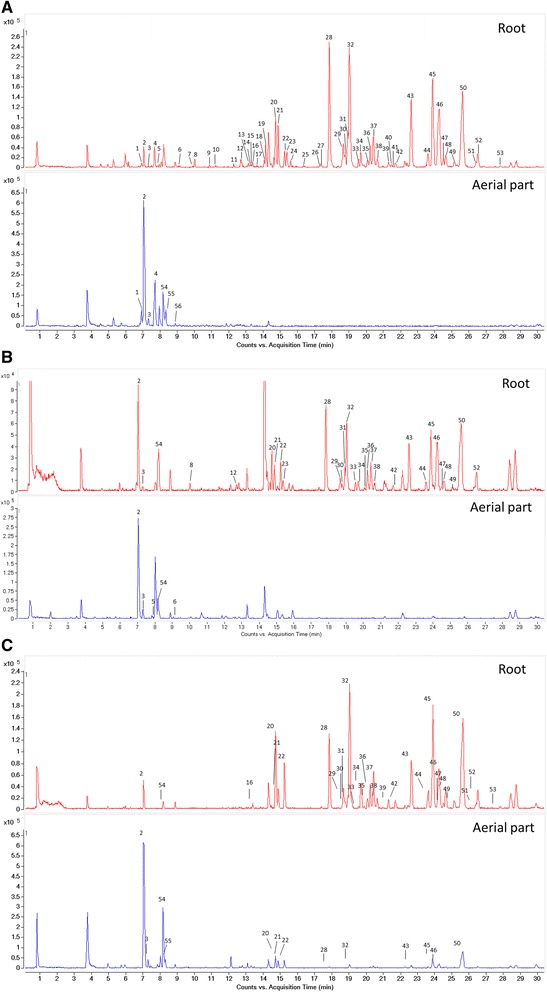
The representative total ions current (TIC) chromatograms of the root and aerial parts from *B. chinense* DC. **a**, *B. yinchowense* Shan et Y. Li **b** and *B. falcatum* L. **c**

The chemical profiles of the root and aerial parts of *Bupleurum* species were analyzed by UHPLC-QTOF-MS. The representative total ions current (TIC) chromatograms of the different parts from *B. chinense*, *B. yinchowense* and *B. falcatum* are shown in Fig. 3. The major peaks in the TIC chromatograms were identified, with peaks 20, 28 and 43 unambiguously identified as saikosaponins c, a and d (SSc, SSa and SSd) by comparison of their chromatographic retention times, accurate molecular weights and characteristic mass fragment ions with those of the references standards. Other peaks were tentatively identified by comparison of their accurate mass data with those reported in the literature. Detailed information related to the illustration of all 56 peaks was shown in Table 2.

**Table 2:** Compounds identified from aerial part and root of three *Bupleurum* species

Peak No.	Identification	t_R_ (min)	Molecular formula	Molecular ions (*m/z*) (mass accuracy (ppm))	Fragments (*m/z*)	BC		BY		BF	
						Root	Aerial	Root	Aerial	Root	Aerial
1	Kaempferol-3-O-glucopyranoside-7-O-rhamnopyranoside	6.90	C_27_H_30_O_15_	593.1537 [M-H]^−^ (4.2)	431.1086 [M-H-(Glc-H_2_O)]^−^, 285.0458 [M-H-(Glc-H_2_O)-(Rha-H_2_O)]^−^	+	+				
2	Rutin	7.03	C_27_H_30_O_16_	609.1439 [M-H]^−^ (−3.61)	1219.3206 [2 M–H]^−^, 300.0334 [M-H-(Glc-H_2_O)-(Rha-H_2_O)]^−^	+	+	+	+	+	+
3	Isoquercitrin	7.30	C_21_H_20_O_12_	463.0871 [M-H]^−^ (−2.4)	300.2900 [M-H-(Glc-H_2_O)]^−^	+	+	+	+		+
4	Kaempferitrin	7.67	C_27_H_30_O_14_	577.1589 [M-H]^−^ (4.5)	431.1079 [M-H-(Rha-H_2_O)]^−^, 285.0477 [M-H-2(Rha-H_2_O)]^−^	+	+	+			
5	Quercetin 3′-glucoside-7-acetat	7.84	C_23_H_22_O_13_	505.0992 [M-H]^−^ (−0.8)	300.0311 [M-2H-Acetyl-(Glc-H_2_O)]^−^	+			+		
6	Quercetin 3,7-diglucoside	9.05	C_27_H_30_O_17_	625.1433 [M-H]^−^ (3.7)	463.1016 [M-H-(Glc-H_2_O)]^−^	+			+		
7	Quercetin 4′-glucoside	9.89	C_21_H_20_O_12_	463.0884 [M-H]^−^ (0.4)	300.283 [M-H-(Glc-H_2_O)]-	+					
8	Unknown	10.01	C_42_H_66_O_15_	809.4367 [M-H]^−^ (4.7) 855.4347 [M + HCOO]^−^ (−4.3)	647.3920 [M-H-(Glc-H_2_O)]^−^	+		+			
9	Rotundifolioside I/Rotundioside O	10.86	C_47_H_76_O_16_	895.5017 [M-H]^−^ (−4.9)	/	+					
10	Rotundioside N	11.23	C_48_H_78_O_18_	941.5142 [M-H]^−^ (2.87) 987.5146 [M + HCOO]^−^ (−2.43)	633.4307 [M-H-(Glc-H_2_O)-(Fuc-H_2_O)]^−^	+					
11	Hydroxy-SSa	12.27	C_42_H_70_O_14_	797.4646 [M-H]^−^ (−5.9) 843.4705 [M + HCOO]^−^ (−5.1)	635.4320 [M-H-(Glc-H_2_O)]^−^	+					
12	Hydroxy-SSd	12.67	C_42_H_70_O_14_	797.4656 [M-H]^−^ (−4.6)) 843.4711 [M + HCOO]^−^ (−4.4)	635.4365 [M-H-(Glc-H_2_O)]^−^	+		+			
13	Rotundioside W	13.12	C_48_H_78_O_18_	941.5195 [M-H]^−^ (8.5) 987.5156 [M + HCOO]^−^ (−1.4)	796.4688 [M-H- (Rha-H_2_O)]^−^	+					
14	Acetyl-hydroxy-SSa	13.22	C_44_H_72_O_15_	839.4793 [M-H]^−^ (−0.6) 885.4824 [M + HCOO]^−^ (−3.28))	797.4801 [M-H-Acetyl]^−^; 635.4276 [M-H-Acetyl-(Glc-H_2_O)]^−^	+					
15	Acetyl-hydroxy-SSd	13.29	C_44_H_72_O_15_	839.4777 [M-H]^−^ (−2.5) 885.4822 [M + HCOO]^−^ (−3.5)	797.4856 [M-H-Acetyl]^−^; 635.4268 [M-H-Acetyl-(Glc-H_2_O)]^−^	+					
16	Malonyl-acetyl-hydroxy-SSa/Malonyl-acetyl-hydroxy-SSd	13.37	C_45_H_72_O_17_	883.4663 [M-H]^−^ (−3.9)	797.4801 [M-H-Malonyl-Acetyl]^−^; 635.4276 [M-H-Malonyl-Acetyl- (Glc-H_2_O)]^−^	+				+	
17	Malonyl-SSn	13.61	C_51_H_80_O_21_	1027.5194 [M-H]^−^ (6.21)	985.5383 [M-H-Acetyl]^−^, 941.5287 [M-H-Acetyl-CO_2_]^−^	+					
18	Unknown	14.06	C_51_H_90_O_26_	1117.5640 [M-H]^−^ (−0.7)	955.5135 [M-H-(Glc-H_2_O)]^−^	+					
19	Unknown	14.15	C_51_H_90_O_26_	1117.5654 [M-H]^−^ (−0.5)	955.5135 [M-H-(Glc-H_2_O)]^−^	+					
20	SSc	14.73	C_48_H_78_O_17_	925.5168 [M-H]^−^ (0.2) 971.5208 [M + HCOO]^−^ (1.3)	779.4734 [M-H-(Rha-H_2_O)]^−^	+		+		+	+
21	SSf	14.87	C_48_H_80_O_17_	927.5313 [M-H]^−^ (1.1) 973.5376 [M + HCOO]^−^ (0.2)	781.4895 [M-H-(Rha-H_2_O)]^−^	+		+		+	+
22	Malonyl-Ssc	15.23	C_51_H_80_O_20_	1011.5162 [M-H]^−^ (−0.8)	967.5447 [M-H-CO_2_]^−^, 779.4838 [M-H-Malonyl-(Rha-H_2_O)]^−^	+		+		+	+
23	Malonyl-Ssf	15.37	C_51_H_82_O_20_	1013.5322 [M-H]^−^ (−0.5)	969.5611 [M-H-CO_2_]^−^, 927.5527 [M-H- Malonyl]^−^, 781.4795 [M-H-Malonyl-(Rha-H_2_O)]^−^, 765.4884 [M-H-Malonyl-(Glc-H_2_O)]^−^	+		+			
24	Malonyl-acetyl-rotundifolioside B	15.59	C_52_H_82_O_21_	1041.5205 [M-H]^−^ (−6.8)	997.5215 [M-H-CO_2_]^−^, 835.4661 [M-H-CO_2_-(Glc-H_2_O)]^−^	+					
25	SSb3 / SSb4	16.34	C_43_H_72_O_14_	811.4847 [M-H]^−^ (−0.3) 857.4985 [M + HCOO]^−^ (0.9)	649.4344 [M-H-(Glc-H_2_O)]^−^	+					
26	SSn	17.31	C_48_H_78_O_18_	941.5196 [M-H]^−^ (0.8) 987.5127 [M + HCOO]^−^ (0.4)	779.4722 [M-H-(Glc-H_2_O)]^−^	+					
27	Malonyl-SSb3 / SSb4	17.38	C_46_H_74_O_17_	897.4823 [M-H]^−^ (3.3)	853.5090 [M-H-CO_2_]^−^, 811.5024 [M-H- Malonyl]^−^, 649.4453 [M-H-Malonyl-(Glc-H_2_O)]^−^	+					
28	SSa	17.83	C_42_H_68_O_13_	779.4558 [M-H]^−^ (−0.4) 825.4629 [M + HCOO]^−^ (−0.2)	617.4186 [M-H-(Glc-H_2_O)]^−^	+		+		+	+
29	Acetyl-SSa	18.65	C_44_H_70_O_14_	821.4668 [M-H]^−^ (−3.0) 867.4713 [M + HCOO]^−^ (−4.0)	779.4742 [M-H-Acetyl]^−^, 617.4236 [M-H-Acetyl-(Glc-H_2_O)]^−^	+		+		+	
30	Acetyl-SSa	18.77	C_44_H_70_O_14_	821.4660 [M-H]^−^ (−4.0) 867.4718 [M + HCOO]^−^ (−3.4)	779.4746 [M-H-Acetyl]^−^, 617.4209 [M-H-Acetyl-(Glc-H_2_O)]^−^	+		+		+	
31	Acetyl-SSa	18.93	C_44_H_70_O_14_	821.4649 [M-H]^−^ (−5.3) 867.4714 [M + HCOO]^−^ (−3.9)	779.4752 [M-H-Acetyl]^−^, 617.4177 [M-H-Acetyl-(Glc-H_2_O)]^−^	+		+		+	
32	Malonyl-SSa	19.01	C_45_H_70_O_16_	865.4576 [M-H]^−^ (−1.7)	821.4851 [M-H-CO_2_]^−^, 779.4747 [M-H- Malonyl]^−^, 617.4182 [M-H- Malonyl-(Glc-H_2_O)]^−^	+		+		+	+
33	Acetyl-SSa	19.55	C_44_H_70_O_14_	821.4657 [M-H]^−^ (−4.4) 867.4713 [M + HCOO]^−^ (−4.0)	779.4746 [M-H-Acetyl]^−^, 617.4188 [M-H-Acetyl-(Glc-H_2_O)]^−^	+		+		+	
34	Dimalonyl-SSa	19.68	C_52_H_72_O_16_	951.4765 [M-H]^−^ (1.8)	907.4863 [M-H-CO_2_]^−^, 779.4687 [M-H-2 Malonyl]^−^, 617.4188 [M-H-2 Malonyl-(Glc-H_2_O)]^−^	+		+		+	
35	Dimalonyl-SSa	20.08	C_52_H_72_O_16_	951.4771 [M-H]^−^ (2.4)	907.4873 [M-H-CO_2_]^−^, 779.4757 [M-H-2 Malonyl]^−^, 617.4244 [M-H-2 Malonyl-(Glc-H_2_O)]^−^	+		+		+	
36	Dimalonyl-SSa	20.22	C_52_H_72_O_16_	907.4874 [M-H-CO_2_]^−^ (2.8)	779.4717 [M-H-2 Malonyl]^−^, 617.4192 [M-H-2 Malonyl-(Glc-H_2_O)]^−^	+		+		+	
37	Dimalonyl-SSa	20.43	C_52_H_72_O_16_	907.4880 [M-H-CO_2_]^−^ (3.4)	779.4764 [M-H-2 Malonyl]^−^, 617.4199 [M-H-2 Malonyl-(Glc-H_2_O)]^−^	+		+		+	
38	Dimalonyl-SSa	20.64	C_52_H_72_O_16_	907.4865 [M-H-CO_2_]^−^ (1.8)	779.4799 [M-H-2 Malonyl]^−^, 617.4156 [M-H-2 Malonyl-(Glc-H_2_O)]^−^	+		+		+	
39	Dimalonyl-acetyl-SSa	21.31	C_54_H_74_O_17_	993.4864 [M-H]^−^ (1.1)	949.4979 [M-H-CO_2_]^−^, 864.5019 [M-2H-Malonyl-Acetyl]^−^, 821.4803 [M-2Malonyl]^−^, 761.4609 [M-H-Malonyl-(Fuc-H_2_O)]^−^	+				+	
40	SSs	21.44	C_59_H_74_O_10_	941.5269 [M-H]^−^ (6.4) 987.5245 [M + HCOO]^−^ (−1.9)	780.4802 [M-(Glc-H_2_O)]^−^ 617.4202 [M-H-2(Glc-H_2_O)]^−^	+					
41	Dimalonyl-SSa / SSd	21.53	C_52_H_72_O_16_	907.4865 [M-H-CO_2_]^−^ (1.8)	779.4799 [M-H-2 Malonyl]^−^, 617.4156 [M-H-2 Malonyl-(Glc-H_2_O)]^−^	+					
42	Malonyl-SSe	21.68	C_45_H_70_O_15_	849.4689 [M-H]^−^ (5.5)	805.4903 [M-H-CO_2_]^−^, 763.4800 [M-H-Malonyl]^−^, 643.4376 [M-H-CO-(Glc-H_2_O)]^−^ 601.4253 [M-H-Malonyl-(Glc-H_2_O)]^−^	+		+		+	
43	SSd	22.62	C_42_H_68_O_13_	779.4547 [M-H]^−^ (−5.1) 825.4606 [M + HCOO]^−^ (−4.4)	617.4186 [M-H-(Glc-H_2_O)]^−^	+		+		+	+
44	Acetyl-SSd	23.60	C_44_H_70_O_14_	821.4653 [M-H]^−^ (−4.9) 867.4756 [M + HCOO]^−^ (0.9)	779.4739 [M-H-Acetyl]^−^, 617.4196 [M-H-Acetyl-(Glc-H_2_O)]^−^	+		+		+	
45	Malonyl-SSd	23.85	C_45_H_70_O_16_	865.4571 [M-H]^−^ (−2.3)	821.4859 [M-H-CO_2_]^−^, 779.4716 [M-H- Malonyl]^−^, 617.4199 [M-H- Malonyl-(Glc-H_2_O)]^−^	+		+		+	+
46	Acetyl-SSd	24.22	C_44_H_70_O_14_	821.4648 [M-H]^−^ (−5.5) 867.4722 [M + HCOO]^−^ (−3.0)	779.4745 [M-H-Acetyl]^−^, 617.4238 [M-H-Acetyl-(Glc-H_2_O)]^−^	+		+		+	+
47	Acetyl-SSd	24.49	C_44_H_70_O_14_	821.4671 [M-H]^−^ (−2.7) 867.4717 [M + HCOO]^−^ (−3.6)	779.4723 [M-H-Acetyl]^−^, 617.4218 [M-H-Acetyl-(Glc-H_2_O)]^−^	+		+		+	
48	Dimalonyl-SSd	24.62	C_52_H_72_O_16_	951.4771 [M-H]^−^ (2.4)	907.4859 [M-H-CO_2_]^−^, 821.4865[M-H- Malonyl-CO_2_]^−^, 779.4799 [M-H-2 Malonyl]^−^, 617.4156 [M-H-2 Malonyl-(Glc-H_2_O)]^−^	+		+		+	
49	Dimalonyl-SSd	25.14	C_52_H_72_O_16_	907.4863 [M-H-CO_2_]^−^ (1.5)	821.4924[M-H- Malonyl-CO_2_]^−^, 779.4829 [M-H-2 Malonyl]^−^, 617.4177 [M-H-2 Malonyl-(Glc-H_2_O)]^−^	+		+		+	
50	Dimalonyl-SSd	25.60	C_52_H_72_O_16_	907.4873 [M-H-CO_2_]^−^ (2.6)	821.4856 [M-H- Malonyl-CO_2_]^−^, 779.4761 [M-H-2 Malonyl]^−^, 617.4191 [M-H-2 Malonyl-(Glc-H_2_O)]^−^	+		+		+	+
51	Dimalonyl-acetyl-SSd	26.40	C_54_H_74_O_17_	993.4828 [M-H]^−^ (−2.5)	949.4978 [M-H-CO_2_]^−^, 821.4838 [M-2Malonyl]^−^	+				+	
52	Dimalonyl-acetyl-SSd	26.52	C_54_H_74_O_17_	993.4896 [M-H]^−^ (4.0)	949.4976 [M-H-CO_2_]^−^, 864.5027 [M-2H-Malonyl-Acetyl]^−^, 821.4860 [M-2Malonyl]^−^, 761.4615 [M-H-Malonyl-(Fuc-H_2_O)]^−^,	+		+		+	
53	Malonyl-diacetyl-SSd	27.81	C_53_H_73_O_15_	949.4971 [M-H]^−^ (1.7)	863.5013 [M-H-Malonyl]^−^, 779.4689 [M-H-Malonyl-2Acetyl]^−^	+				+	
54	Isorhamnetin-3-O-rutinoside	8.20	C_28_H_32_O_16_	623.1651 [M-H]^−^ (5.3)	/		+	+	+	+	+
55	Quercetin-3-O-rhamnoside	8.32	C_21_H_20_O_11_	447.0936 [M-H]^−^ (0.7)	300.0349 [M-H-(Rha-H_2_O)]^−^		+				+
56	Quercetin 3-(6″-acetylglucoside)	8.54	C_23_H_22_O_13_	505.0993 [M-H]^−^ (1.0)	300.0330 [M-2H-Acetyl-(Glc-H_2_O)]^−^		+				

In the TIC chromatograms, saikosaponins and flavonoids represents majority of the peaks identified, with flavonoids (peaks 1, 2, 3, 4, 5, 6, 7, 54, 55 and 56) accumulated in the previous 10 min of the eluting time while saikosaponins were eluted at the rest of time. The root and aerial parts of all the three *Bupleurum* species exhibited varied profiles. It is distinct and consistent for all the roots contain more chromatographic peaks covering both of the saikosaponin and flavonoid part of the TIC chromatograms. The profiles of the roots from the three *Bupleurum* species were similar especially for the characteristic saikosaponin part (peaks 20–52). In contrast, the aerial parts contain abundantly flavonoids with little or no saikosaponin peaks. The results clearly demonstrated the distinct chemical profiles of the root and aerial parts of *Bupleurum* species. Yen et al. compared the saikosaponins a, c and d between the root and aerial parts of three *Bupleurum* species using thin-layer chromatography (TLC) scanning. The results showed that the aerial parts contained low levels of saikosaponins, which were different from that of the root [24]. These were in accordance with those provided in the present study, which thus strengthened the conclusions that the aerial parts could not be used as an alternate of root from a chemodiversity perspective.

### Principal component analysis (PCA)

**Fig. 4:**
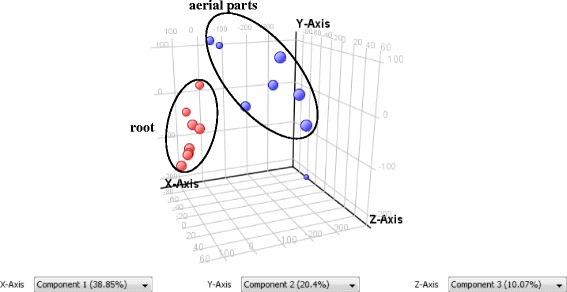
The principal component analysis (PCA) of the root (*red*) and aerial (*blue*) parts from eight batches of *Bupleurum* samples

The differences between the root and aerial parts of eight herbal samples were further displayed by the Principal Component Analysis (PCA). The full time-of-flight (TOF) mass spectral data of each sample were first processed by MassHunter Workstation software. Ions were extracted by molecular feature extraction (MFE) algorithm characterized by retention time (RT), intensity in apex of chromatographic peak, and accurate mass, exported as the Compound Exchange Format (.cef) file. These results were then analyzed by Mass Profiler Professional (MPP) software. Entities that present in more than 50% of samples in at least one condition were filtered by frequency before doing Principal Component Analysis (PCA). Finally 258 features were left for further PCA study. The resulting PCA graph also demonstrated the distinct clustering between the root and aerial parts of the investigated samples, which indicating the chemical difference between these two parts (Fig. 4).

### Potential differentiating markers

In order to find out the potential differentiating markers for distinguishing the different parts of *Bupleurum* species, significant testing and fold change was investigated to identify statistically differentiative compounds by applying appropriate test and conditions. Nine compounds, out of 258 entities from the above frequency filtration were found to be significantly different among the two parts using one way ANOVA and a level of probability of 0.001 and fold change >2, as listed in Table 3. These differentiating markers with the lowest *p*-values and highest fold-changes (most significant with greatest abundance differences) posed mostly influential features for the differentiation between the root and aerial parts, which therefore could be used as markers for differentiation.

**Table 3:** List of compounds (9 entities) that are distinguished between root and aerial parts at *p* < 0.001 and fold change (FC) > 3

Peak No.	Compounds	R.T. (mins)	*p*-value	Log FC
3	Isoquercitrin	7.30	2.09 × 10^−6^	−15.798277
23	Malonyl-Ssf	15.37	7.41 × 10^−6^	15.704748
28	Saikosaponin a	17.83	6.74 × 10^−6^	17.776505
32	Malonyl-Ssa	19.01	6.61 × 10^−6^	16.194246
43	Saikosaponin d	22.62	7.21 × 10^−6^	17.776505
44	Acetyl-Ssd	23.60	7.90 × 10^−6^	15.572599
45	Malonyl-Ssd	23.85	1.33 × 10^−16^	20.250618
46	Acetyl-Ssd	24.22	6.35 × 10^−6^	17.99062
52	Dimalonyl-acetyl-Ssd	26.52	6.61 × 10^−6^	16.017168

## Conclusions

The present study revealed the distinct chemical profiles between the root and aerial parts of *Bupleurum* species, which indicated that the aerial parts could not be used as an alternative of root from a chemodiversity perspective. Meanwhile, the established UHPLC-QTOF-MS method in the present study as well as the potential differentiating markers could be utilized to profile and distinguish the root and aerial parts of *Bupleurum* species from different species or locations. Thus, the approach established here will provide a comprehensive analysis of chemical profiles between the root and aerial parts from the commonly used *Bupleurum* species which will be helpful for testing the crude materials of proprietary Chinese medicines containing Chaihu as well as for establishing guidelines for the appropriate clinical use of Chaihu. Furthermore, this information will be of great significance to the efficient use of botanical resources.

## Abbreviations

MFE: Molecular feature extraction
PCA: Principal component analysis
RT: Retention time
SSa: Saikosaponin a
SSc: Saikosaponin c
SSd: Saikosaponin d
SSs: Saikosaponins
TCM: Traditional Chinese medicine
TIC: Total ions current
TLC: Thin-layer chromatography
UHPLC-QTOF-MS: Ultra-high performance liquid chromatography quadrupole/time of flight-mass spectrometry
